# Exogenous application of selenium and nano-selenium alleviates salt stress and improves secondary metabolites in lemon verbena under salinity stress

**DOI:** 10.1038/s41598-023-32436-4

**Published:** 2023-04-01

**Authors:** Fardin Ghanbari, Majid Bag-Nazari, Afsaneh Azizi

**Affiliations:** 1 Department of Horticultural Science, Faculty of Agriculture, Ilam University, Ilam, Iran; 2 Department of Horticultural Science, Faculty of Agriculture, Shahid Chamran University, Ahvaz, Iran

**Keywords:** Analytical biochemistry, Biochemistry, Biotechnology, Nanoscience and technology, Plant sciences, Plant biotechnology, Plant physiology, Plant stress responses, Secondary metabolism

## Abstract

Salinity stress is one of the most serious abiotic factors that affects the growth, performance, and secondary compounds of medicinal plants. The aim of this study was to determine the effect of foliar application of selenium and nano-selenium separately on growth, essential oils, physiological parameters, and some secondary metabolites of Lemon verbena under salinity stress. The results showed that selenium and nano-selenium significantly increased growth parameters, photosynthetic pigments, and relative water content. Compared to the control, a higher accumulation of osmolytes (i.e., proline, soluble sugars, and total protein) and higher antioxidant activity were observed in the selenium-treated plants. In addition, selenium alleviated the adversary effect of oxidative stress, resulting from salinity, by reducing leaf electrolyte leakage, malondialdehyde, and H_2_O_2_ accumulation. Furthermore, selenium and nano-selenium enhanced the biosynthesis of secondary metabolites such as essential oils, total phenolic content, and flavonoid compounds under non-stress and salinity conditions. They also reduced Na^+^ accumulation in the root and shoot of the salinity-treated plants. Hence it can be concluded that exogenous application of selenium and nano-selenium separately can mitigate the negative effects of salinity by improving the quantitative and qualitative performance of lemon verbena plants under salinity stress.

*Aloysia citrodora* Paláu (*Lippia citriodora* Kunth), known as lemon verbena, belongs to Verbenaceae family. As a medicinal plant native to South America, North Africa and Southern Europe^6^, it is extensively used in folk medicine because of its antispasmodic, antipyretic, antimalarial, anti-inflammatory, sedative, and digestive properties^37^. Due to its extensive applications in different industries and folk medicine, the cultivation of lemon verbena in Iran has been expanded^21^.

Salinity is one of the most severe environmental factors that can reduce the growth and productivity of plants^56^. It has been estimated that worldwide, 20% of all cultivated land and 33% of irrigated agricultural land are exposed to high salinity, and saline land is increasing yearly^47^. In Iran, estimates indicate that about 34 million hectares, including 1.4 million hectares of irrigated lands, are under salinity^41^. The accumulation of different salts in the rhizosphere leads to competition for the absorption of nutrients, reduces the absorption of essential elements and the accumulation of toxic elements, such as chlorine and sodium in plants^24,45,54^. Also, the presence of different salts in the soil increases the osmotic potential of the soil solution and makes it difficult for plants to absorb water, which is called the osmotic effect of salinity^34^. Therefore, salinity affects the growth and performance of plants by disrupting the absorption of nutrients and water.

Plant nutrition is one of the most effective ways to reduce the effects of oxidative stress in plants^31^. Selenium (Se) is an essential trace element for humans with respect to thyroid hormone metabolism, antioxidant defense, anti-inflammatory and immune activities^44^. So far, this element is not known to be essential for plants,however, numerous researchers have revealed the effects of Se on growth and development of plants under stress and non-stress conditions^11,13,32,43^. Kong et al.^30^ reported that the application of Se, preserves the ultra-structures of chloroplasts and mitochondria, and as a result, by improving plant photosynthesis, it increases tolerance to salt stress in sorrel plants. Se treatment also protected the cell membrane and increased the cadmium tolerance of cucumber (Hawrylak-Nowak, 2014). Soleymanzadeh et al.^50^ reported that the use of Se nanoparticles treatment in strawberry plant under salinity stress led to an increase in the concentration of salicylic acid, improved the homeostasis of ions, thereby increasing the antioxidant activity and ultimately stress tolerance in this plant. The positive effect of Se in plants is related to the regulation of antioxidant defense systems and photosynthesis^25^.

Nanoparticles are atomic or molecular assemblies with minimum dimensions between 1 and 100 nm, which have different physicochemical properties than their bulk materials^26^. In fact, the conversion of materials to the Nano-scale changes their mechanical, chemical, electrical, and optical properties and contributes to penetration power into cell membranes^12^. Some researchers have reported the positive effect of Nano-materials on the growth and development of plants under stress and non-stress conditions^50,55^, while there is no report on similar effects in lemon verbena plants. Therefore, in the present research, the separate effect of Se and N-Se on salinity stress tolerance of lemon verbena plants has been investigated. Our hypothesis was that Se and N-Se could alleviate the effects of salinity stress on lemon verbena plants due to their antioxidant effects. Therefore, in this research, the effects of Se treatments and salinity stress on growth parameters, RWC, osmolytes accumulation, MDA, H_2_O_2_, ion concentrations, antioxidant enzymes, and secondary compounds of lemon verbena plants have been evaluated.

## Materials and methods

### Chemicals

Nano-selenium (N-Se) used in this research was obtained from NANOSANY (Mashhad, Iran). The properties of these Nano-materials have already been described^55^. Also, Na_2_SeO_3_ prepared from Sigma-Aldrich was used as a Se source.

### Plant materials and treatments

This experiment was conducted in the Faculty of Agriculture, Ilam University, Ilam province, Iran. Rooted lemon verbena cuttings were purchased from a commercial unit affiliated with the Agricultural Research Center and Natural Resources of Ilam province. The cuttings (1 plant/pot) were planted in 4-L volume plastic pots filled with an equal mixture of coco-peat and perlite. The pots were kept in a greenhouse under ambient environmental conditions with temperature regime 25/18 ± 2 °C day/night and relative humidity of 60–70%, for one month. All the pots were watered with 1/4 Hoagland solution every three days, and after full establishment of conditions, experimental treatments were started. Three Se treatments (control (distilled water), 10 μM N-Se, and 10 μM Se) were used in combination with five salinity treatments (control, 40, 80, 120, and 160 mM NaCl). The NaCl treatments in the mentioned concentrations were prepared fresh and added to the nutrient solution, and a part of the solution was drained from the pots. Se treatments were applied one week after starting salinity treatment as foliar spray until runoff and repeated every two weeks. Based on a preliminary experiment, lemon verbena plants treated with 10 μM Se showed the best growth under salinity stress (100 mM NaCl), so this concentration were chosen for the main experiment (data not shown). The treatments affected the plants for two months, and before the full flowering stage, the samples were collected, and the fresh and dry weights of the roots and shoots were measured immediately. The leaf samples were dried in the shade and used to measure the essential oil content. Also, for phytochemical measurements, fresh samples were collected and immediately frozen in liquid N_2_, stored at − 80 °C for further analyses.

### Plant analysis

#### Pigments and relative water content

Plant pigments, including chlorophyll a, chlorophyll b, and total chlorophyll, were extracted according to the method of Arnon et al.^4^ using 80% acetone solvent. The absorbance of the samples was read at wavelengths of 663 and 645 nm using a spectrophotometer (Specord 50, Analytik Jena), and the content of chlorophylls was reported based on mg/g of leaf fresh weight.

The relative water content (RWC) was determined according to Alfosea-Simón et al.^3^ method. First, 1 cm leaf discs were separated from each plant. The samples were weighed using a digital scale (FW) and then kept for 16 h in distilled water (for complete turgor) and then weighed again after drying the surface water (TW). After that, the leaves were placed in an electric oven at a temperature of 60 °C for 24 h. Then the leaves were weighed to obtain dry weight (DW). The RWC of the leaf was calculated from the following equation:

$$Relative\,water\,content = \frac{FW - DW}{{TW - DW}} \times 100$$

### Electrolyte leakage, malondialdehyde and H_2_O_2_ accumulation

Leaf electrolyte leakage (LEL) was measured according to the method of Lutts et al.^33^. For this purpose, 10 leaf discs were randomly separated from the plants of each treatment, and after washing, they were transferred to special vials. 10 ml of distilled water was added to the vials and placed on a shaker at a speed of 150 rpm for 24 h at room temperature. The electrical conductivity of the solution was read using an EC meter (EC1). Then the samples were placed inside the autoclave at 121 °C for 20 min. After cooling down at room temperature, the second reading of the solution (EC2) was done, and based on the following equation, the LEL was calculated and reported as a percentage.

$$Leaf\,elecrolyte\,leakage = \frac{EC1}{{EC2}} \times 100$$

The amount of peroxidation of membrane lipids was measured using malondialdehyde (MDA) measurement. 200 mg of fresh leaf sample was extracted using 5 mL of 0.1% trichloroacetic acid (TCA) solution. After centrifugation, 1 mL of supernatant was mixed with 1 mL of 0.5% thiobarbituric acid solution dissolved in 20% TCA. The reaction mixture was heated at 95 °C for 30 min. After cooling the mixture and centrifugation (Labnet Prism R) at 10000 g for 15 min, the absorbance of the samples was read at wavelengths of 532 and 600 nm^51^. The amount of H_2_O_2_ was also measured based on the reaction with KI^2^.

### Osmotic regulating compounds

Proline and soluble sugar content were measured in fresh leaf samples according to Bates et al.^7^ and Irigoyen et al.^22^. Also, protein content was spectrophotometrically estimated based on the method of Bradford^9^.

### Activity of antioxidant enzymes

To measure enzyme activity, 200 mg of frozen leaf tissue was extracted in 2 ml of 50 mM sodium phosphate buffer containing 1 mM EDTA, 50 mM Tris–HCl, and 2% Polyvinylpyrrolidone (PVP). After centrifugation (Labnet Prism R) at a 10,000 rpm at 4 °C for 15 min, the supernatant was collected and used as crude enzyme extract to measure enzyme activity. Peroxidase activity (POD) was performed according to the method of Plewa et al.^39^. The reaction mixture included 100 µL of enzyme extract, 2.5 mL of sodium phosphate buffer, 100 µL of H_2_O_2_, and 100 µL of guaiacol. The absorbance of the sample was read for 2 min at a wavelength of 470 nm, and the enzyme activity was reported based on enzyme units (U) per milligram of protein. Catalase enzyme activity (CAT) using the method of Dhindsa et al.^14^ measured at a wavelength of 240 nm. Also, the activity of the superoxide dismutase (SOD) enzyme was measured through inhibition of nitro blue tetrazolium (NBT) photo-reduction and formazan formation^16^.

### Total phenol content, total flavonoid content and total antioxidant capacity

The Folin–Ciocalteu method was used to measure the amount of total phenol^48^. For this purpose, 200 mg of leaf sample was pounded by adding 3 mL of 85% methanol, and after filtering, 300 µL of supernatant was added to 1500µL of 10% Folin regent, and it was placed at room temperature for 5 min. Then 1200 µL of 7% sodium carbonate were added to it. After two hours of shaking on the shaker at room temperature, the absorbance of the solution was measured at a wavelength of 765 nm using a spectrophotometer. The result was expressed as mg GAE/g FW.

Total flavonoid content was determined according to the method of Skrypnik et al.^49^ with slight modifications. 100 µL of 5% sodium nitrite and 50 µL of AlCl_3_ were added to 1 mL of sample extracts. After 10 min, 250 μl of 1 M NaOH was added to the reaction mixture. The absorbance of the solution was read at a wavelength of 510 nm using a spectrophotometer. Finally, the flavonoid content was calculated based on the quercetin standard curve, and the results were reported as mg/g FW. Also, total antioxidant capacity (TAC) was measured using the DPPH method described by Brand-Williams et al.^10^.

### Essential oil extraction

The extraction and measurement of essential oil percentage in lemon verbena were done using the method of Hosseini et al.^21^. For this, lemon verbena leaves were placed in the shade at room temperature for 14 days to dry. Extraction of the essential oil of dried leaves was done by distillation with water for 3 h using a Clevenger device. Based on the initial dry weight, essential oil content (%) and essential oil yield (mL/pot) were calculated for each experimental unit.

### Elemental analysis

For the analysis of mineral nutrients, leaf and root samples were ground separately and ashed at 550 °C for 4 h. 100 mg of the prepared ash was dissolved with 5 ml of 2N HCl and then made up to 50 mL with distilled water. The Na^+^ concentration of leaf and root samples was measured using a film photometer, and Cl^−^ concentration was also measured by silver nitrate titration^15^.

### Statistical analysis

A factorial experiment was arranged in a completely randomized design with three replicates. The investigated factors were salinity stress and selenium application. Statistical analysis was carried out with SAS version 9.1 software (SAS Institute Inc., Cary, NC, USA). Data were analyzed with analysis of variance (ANOVA) and the treatment means were compared using Duncan's multiple range test (*p* < 0.05).

### Ethical approval

Experimental research on plants in this study, including the collection of plant material, complies with relevant institutional, national, and international guidelines and legislation.

## Results

### Plant growth

In order to evaluate the effect of salinity and Se treatments on the growth rate of lemon verbena plants, some plant growth indicators, including plant height, shoot fresh weight (SFW), shoot dry weight (SDW), root fresh weight (RFW) and root dry weight (RDW) were measured. The results showed that the salinity × Se interaction influenced all the mentioned growth parameters (Table 1). With increasing salinity levels, the growth parameters decreased significantly, so the lowest growth traits were observed at the most severe level of salinity stress (160 mM NaCl). Using N-Se and Se reduced the effects of salinity stress on plant growth parameters, and at all salinity levels, it improved lemon verbena plant growth compared to the control. In other words, an enhancement in the plant height (18.57, 7.05, 3.79 ns, 11.07, and 20.54%), SFW (46.89, 87.27, 106.24, 131.79, 126.29%), SDW (47.21, 116.92, 113.86, 113.07 and 122.60%), RFW (32.81, 62.16, 86.22, 109.72 and 131.46%) and RDW (80.68, 102.66, 57.20, 66.43 and 32.31%, respectively) were observed in plant treated by 10 µM N-Se subjected to 0, 40, 80, 120 and 160 mM NaCl, as compared with control plants. Likewise, Compared with untreated control plants, plants treated by 10 µM Se displayed notable enhancement in plant height (18.03, 12.71, 9.49, 14.74 and 30.35%), SFW (22.24, 53.15, 72.13, 100.29 and 103.31%), SDW (17.04, 91.38, 89.05, 75.59, 63.61%), RFW (13.40, 40.03, 63.41, 88.39 and 106.72%) and RDW (22.84, 49.83, 45.30, 53.06 and 18.29 ns %), respectively (Table 2).

**Table 1:** Summary of the ANOVA for variables traits in lemon verbena (*Lippia citriodora*) plants subjected to salinity levels (0, 40, 80, 120 and 160 mM NaCl) and selenium (Control, 10 µM selenium and 10 µM nano-selenium) treatments.

traits	Selenium	Salinity	Selenium × Salinity
Plant height	**	**	*
Shoot fresh weight	**	**	*
Shoot dry weight	**	**	**
Root Fresh Weight	**	**	*
Root dry weight	**	**	**
Relative water content	**	**	ns
Chlorophyll a	**	**	**
Chlorophyll b	**	*	**
Total chlorophyll	**	**	**
Electrolyte leakage	**	**	ns
Malondialdehyde	**	**	**
Hydrogen peroxide	**	**	**
Proline	**	**	ns
Protein	**	**	ns
Soluble sugar	**	**	**
CAT activity	**	**	*
POD activity	ns	**	**
SOD activity	**	**	*
Total phenol	**	**	*
Flavonoids	**	**	*
Total antioxidant capacity	**	**	ns
Essential oil percentage	**	**	**
Essential oil yield	**	**	**
Root sodium content	**	**	**
Shoot sodium content	*	**	*
Root chlorine content	ns	**	ns
Shoot chlorine content	**	**	ns

ns: no significant difference.

*Significant difference at *P* < 0.5.

**Significant difference at *P* < 0.01.

**Table 2:** The interactive effect of salinity and selenium treatments on morphological traits in lemon verbena (*Lippia citriodora*). Se: selenium, N-Se: nano-selenium.

Salinity stress (mM)	Selenium	Plant height (cm)	Shoot fresh weight (g)	Shoot dry weight (g)	Root fresh weight (g)	Root dry weight (g)
0	Control	61.00 cd	44.82 cd	16.90d	38.79d	8.23de
	10uM Se	72.33a	65.84a	24.88a	51.25a	14.87a
	10uM N-Se	72.00a	54.79b	19.78bc	43.99bc	10.11c
40	Control	59.00d	31.66gh	9.75 fg	29.10gh	6.00gh
	10uM Se	63.16bc	59.29b	21.15b	47.19b	12.16b
	10uM N-Se	66.50b	48.49c	18.66c	40.75 cd	8.99d
80	Control	52.66 fg	20.67jk	6.85i	20.17j	4.79j
	10uM Se	54.66ef	42.63de	14.65e	37.57de	7.53ef
	10uM N-Se	57.66de	35.58 fg	12.95e	32.96 fg	6.96f.
120	Control	45.16i	16.73kl	6.35ij	16.55j	4.41j
	10uM Se	50.16gh	38.78ef	13.53e	34.71ef	7.23f.
	10uM N-Se	51.83fgh	33.51 fg	11.15f.	31.18 fg	6.75 fg
160	Control	37.33j	11.75 l	4.70j	11.60 k	4.24j
	10uM Se	45.00i	26.59hi	9.06gh	26.85hi	5.61hi
	10uM N-Se	48.66 h	23.89ij	7.69hi	23.98i	5.02ij

Different lower case letters within the same column indicate significant differences at *p* < 0.05, according to Duncan’s multiple range test. Data represent means of three replication.

### Pigments

The changes in plant pigments in lemon verbena under salinity stress and Se foliar application are presented in Table 3. In comparison with non-stressed control plants, the contents of chlorophyll a, chlorophyll b and total chlorophyll markedly decreased by 20.00, 12.50 and 15.87% in 40 mM NaCl, 32.50, 33.92 and 32.53% in 80 mM NaCl, 25.41, 42.85, 42.00% in 120 mM NaCl and 45.00, 44.64 and 44.44% in 160 mM NaCl, respectively. Lemon verbena plants under foliar application with N-Se and Se at all levels of salinity stress showed improvement in photosynthetic pigments compared to the control. In normal condition, the application of 10 μM N-Se and Se significantly increased chlorophyll a (23.75 and 8.75%), chlorophyll b (28.57 and 7.14%) and total chlorophyll (26.19 and 8.73%) compared to the control. Likewise, N-Se and Se treatments caused an increase of 18–34% in chlorophyll a, 12–38% in chlorophyll b (except for N-Se treatment in 40 mM salinity) and 6–34% in total chlorophyll in plant leaves under salt stress compared to the control (Table 3).

**Table 3:** The interactive effect of salinity and selenium treatments on pigments, malondialdehyde and hydrogen peroxide in lemon verbena (*Lippia citriodora*).

Salinity (mM)	Selenium	Chlorophyll a (mg/g FW)	Chlorophyll b (mg/g FW)	Total chlorophyll (mg/g FW)	Malondialdehyde (nmol/g FW)	Hydrogen peroxide (µmoll/g FW)
0	Control	0.80bc	0.56bc	1.26bc	1.47 g	2.70e
	10uM Se	0.99a	0.72a	1.59a	1.81 fg	2.77e
	10uM N-Se	0.87b	0.60b	1.37b	1.62 g	3.05de
40	Control	0.64efgh	0.49bcde	1.06def	1.63 g	3.56de
	10uM Se	0.75cde	0.46cde	1.13def	1.87 fg	3.64de
	10uM N-Se	0.76bcd	0.55bcd	1.22bcd	1.93 fg	2.63e
80	Control	0.54hi	0.37efg	0.85ghi	2.89 cd	5.68bc
	10uM Se	0.66defg	0.45cdef	1.04defg	1.95 fg	2.89de
	10uM N-Se	0.67def	0.42defg	1.02efg	1.99 fg	2.24e
120	Control	0.47i	0.32 fg	0.73hi	3.50b	7.40b
	10uM Se	0.63fgh	0.43cdefg	0.98efg	3.03bcd	6.20bc
	10uM N-Se	0.61fgh	0.39efg	0.93 fg	2.28ef	4.64 cd
160	Control	0.44i	0.31 g	0.70i	4.12a	10.16a
	10uM Se	0.53hi	0.41efg	0.88fghi	3.38bc	6.35bc
	10uM N-Se	0.55ghi	0.43cdefg	0.91fgh	2.56de	6.27bc

Se: selenium, N-Se: nano-selenium.

Different lower case letters within the same column indicate significant differences at *P* < 0.05, according to Duncan’s multiple range test. Data represent means of three replication.

### Oxidative damage

Electrolyte leakage, malondialdehyde accumulation, and H_2_O_2_ were measured as criteria of oxidative damage caused by salinity stress in lemon verbena plants. The main effect of salinity and Se on LEL, MDA, and H_2_O_2_ was significant (Table 1). With the increase of salinity stress, the content of LEL increased so that the lowest (47.07%) was obtained in the control treatment and the highest (69.65%) in the 160 mM NaCl treatment. The LEL in leaves of plants treated with 10 μM N-Se and Se was 14.55 and 8.86% lower than those treated with distilled water, respectively (Table 4). The content of MDA and H_2_O_2_ were affected by the salinity × Se interaction (Table 1). Compared with control plants, accumulation of MDA (11–180%) and H_2_O_2_ (32–285%) were observed in salinity-stressed plants. At 0 and 40 mM NaCl levels, Se and N-Se treatments had no significant effect on the accumulation of MDA and H_2_O_2_ in lemon verbena plants compared to the control. Interestingly, at the levels of 80, 120, and 160 mM NaCl, the application of N-Se (32.52, 34.58, and 37.86%, respectively) and Se (31.14, 34.85, and 17.96%, respectively) caused a significant decrease in the accumulation of MDA compared to the control. In the same way, the use of N-Se (60.56, 37.29, and 38.28%, respectively) and Se (49.11, 16.21, and 37.50%, respectively) caused a decrease in the accumulation of H_2_O_2_ at salinity levels of 80, 120 and 160 mM, compared to control (Table 3).

**Table 4:** The results of comparing the means of the simple effects of salinity and selenium treatments on relative water content, electrolyte leakage, proline and protein content in lemon verbena plants (*Lippia citriodora*).

Treatments	Relative water content (%)	Electrolyte leakage (%)	Proline (µmol/g FW)	Protein (mg/g FW)
Salinity (mM)				
0	83.60a	47.07e	10.58d	8.41a
40	82.44ab	52.49d	11.44d	7.45b
80	82.27ab	57.96c	15.72c	7.18bc
120	80.54a	63.86b	17.96b	6.68 cd
160	77.86c	69.65a	20.50a	6.15d
Selenium				
Control	76.02c	63.14a	13.31c	6.36c
10uM Se	86.22a	53.95c	16.87a	7.93a
10uM N-Se	81.80b	57.54b	15.53b	7.24b

Se: selenium, N-Se: nano-selenium.

Different lower case letters within the same column indicate significant differences at *P* < 0.05, according to Duncan’s multiple range test. Data represent means of three replication.

### Relative water content

The results showed that the main effects of salinity and Se on RWC were significant, but the interaction effect of salinity × Se on RWC was not significant (Table 1). Salinity stress up to the level of 80 mM NaCl had no significant effect on the RWC of lemon verbena leaves, but the salinity of 120 and 160 mM NaCl decreased the RWC by 3.62 and 6.86%, respectively, compared to the control. On the other hand, the leaves of plants treated with N-Se and Se showed a higher RWC of 13.41% and 7.60%, respectively, than the control plants (Table 4).

### Osmolyte accumulation

The results of ANOVA showed that the effects of salinity and Se on proline and total protein content were significant (Table 1). Salinity stress significantly increased proline (8–94%) and decreased total protein (11–26%) compared to normal conditions. On the other hand, plants treated with N-Se and Se showed an increase of 26.74 and 16.67% of proline and 24.68 and 13.83% of total protein, respectively (Table 4). The effect of salinity, Se, and their interaction on soluble sugar was significant (Table 1). In the absence of Se application, salinity stress at 40, 80, 120 and 160 mM levels caused an increase of 13.70, 62.50, 79.83, and 78.62% of soluble sugar compared to the control, respectively. On the other hand, the results showed that N-Se and Se treatments at all salinity levels improved the soluble sugar (between 15 and 41%) compared to the control. The most significant effect of Se treatments on soluble sugar was observed at the highest salinity level (160 mM), so the treatment of 10 µM N-Se caused a 41.53% increase in soluble sugar compared to the control (Table 5).

**Table 5:** The interactive effect of salinity and selenium treatments on soluble sugar, total phenol and flavonoids in lemon verbena (*Lippia citriodora*).

Salinity (mM)	Selenium	Soluble sugar (mg/g FW)	Total phenol (mg/g FW)	Flavonoids (mg/g FW)
0	Control	7.44i	6.34 g	0.59 h
	10uM Se	10.23 g	6.98ef	0.85f.
	10uM N-Se	10.29 g	6.50 fg	0.78 fg
40	Control	8.46 h	7.18cde	0.70gh
	10uM Se	11.49f.	7.30bcde	0.91ef
	10uM N-Se	11.43f.	7.74ab	1.01de
80	Control	12.09f.	6.94ef	0.84f.
	10uM Se	14.79d	7.56abcd	1.11 cd
	10uM N-Se	13.92e	7.65abc	1.12bc
120	Control	13.35e	7.08de	1.03de
	10uM Se	17.10b	7.60abc	1.22bc
	10uM N-Se	15.30 cd	8.06a	1.41a
160	Control	13.29e	7.47bcd	1.24bc
	10uM Se	18.81a	7.57abcd	1.27b
	10uM N-Se	16.14c	7.71ab	1.40a

Se: selenium, N-Se: nano-selenium.

Different lower case letters within the same column indicate significant differences at *P* < 0.05, according to Duncan’s multiple range test. Data represent means of three replication.

### Antioxidant enzymes

**Figure 1:**
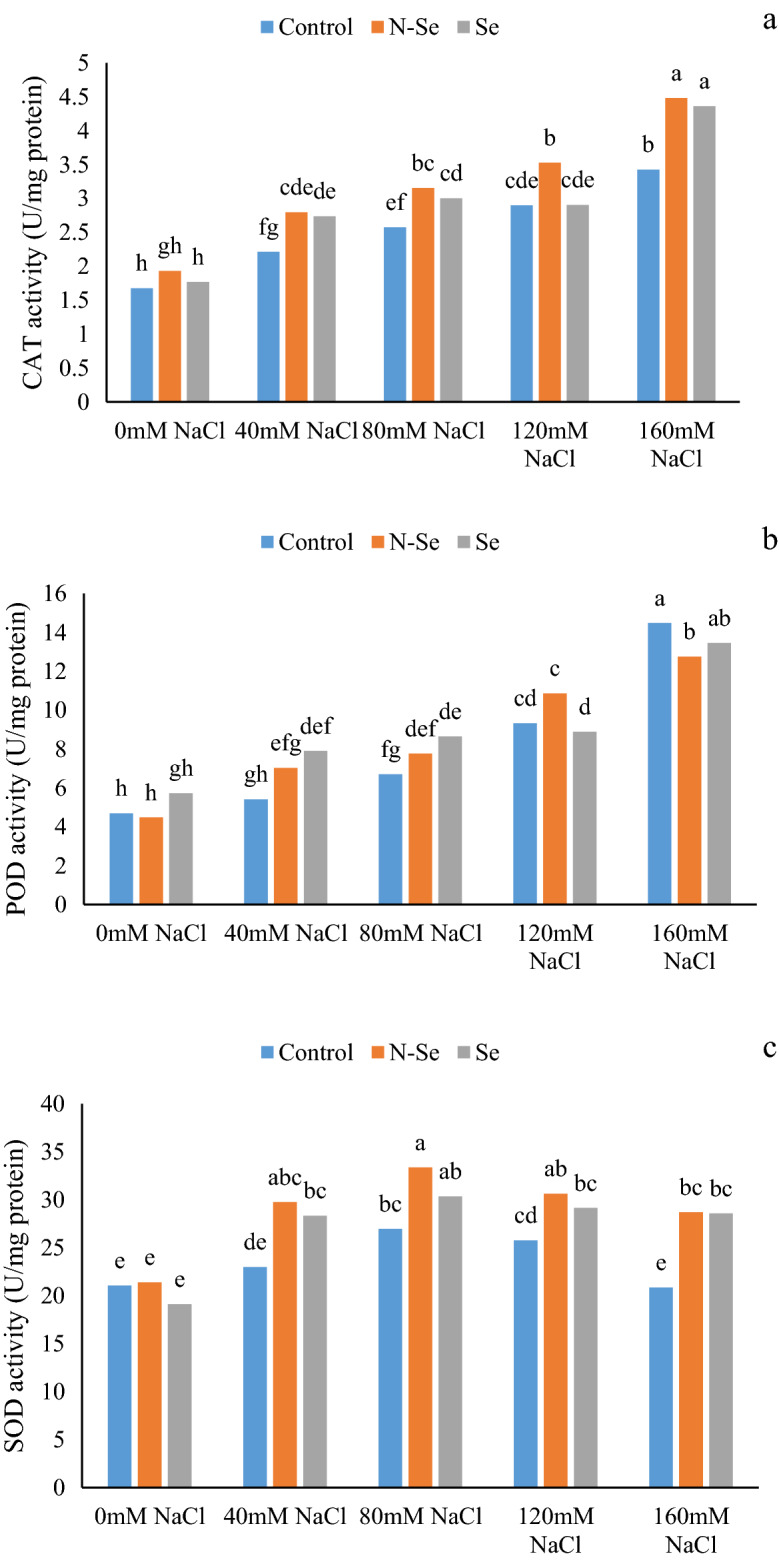
The influence of foliar sprays of selenium (Se) and nano-selenium (N-Se) on CAT (**a**), POD (**b**) and SOD (**c**) enzyme activities of lemon verbena (*Lippia citriodora*) plants under different salinity levels. Data represent means of three replication. Different letters in each column indicate significant differences at *p* < 0.05, according to Duncan’s multiple range test.

The results showed that the activity of antioxidant enzymes, including CAT, POD, and SOD, was significantly affected by the interaction of salinity and Se treatments (Table 1). In control plants (without Se application), salt stress at 40, 80, 120 and 160 mM NaCl levels caused a significant increase of 32.33, 53.89, 73.05 and 104.79% of CAT activity compared to compared to stress-free control plants (Fig. 1a). Likewise, the activity of the POD improved by 40.81, 99.14, and 209.40% in salinity treatments of 80, 120, and 160 mM, respectively, compared to stress-free control plants (Fig. 1b). Furthermore, in control plants, only 80 and 120 mM NaCl treatments improved (28.01 and 22.45%, respectively) the activity of the SOD enzyme compared with the non-stressed plants (Fig. 1c). In general, N-Se and Se treatments had no significant effect on the activities of catalase, peroxidase, and superoxidase enzymes in salt-free conditions (Fig. 1). At the levels of 40, 80, 120 and 160 mM NaCl the application of N-Se (26.22, 22.50, 22.14 and 30.99% respectively) and Se (23.52, 16.73, 0.34and 27.48% respectively) increased the activity of CAT enzyme compared to the control (Fig. 1a). N-Se treatment had no significant effect on POD enzyme activity (except at 160 mM salinity) compared to the control. At salinity levels of 40 and 80 mM NaCl, the application of Se increased the POD activity by 46.11 and 29.14%, respectively, compared to the control (Fig. 1b). Application of 10 μM N-Se at salinity levels of 40, 80, 120, and 160 mM NaCl increased the activity of the SOD enzyme by 29.46, 23.77, 18.90, and 37.64%, respectively, compared to the control. Similarly, the activity of this enzyme increased by 23.28, 12.50, 13.12, and 36.97% compared to the control with the application of 10 μM Se at the mentioned salinity levels (Fig. 1c).

### Total phenol, flavonoid and total antioxidant capacity

The results showed that the effects of salinity, Se, and the interaction effect of salinity × Se on TPC and flavonoids were significant (Table 1). Compared with untreated control plants, enhancement in the TPC (13.24, 9.46, 11.67 and 17.82%), and total flavonoid (18.64, 42.37, 74.57, and 110.16%) were observed in plant treated with 40, 80, 120 and 160 mM NaCl, respectively. On the other hand, the results showed that the plants treated with 10 μM N-Se at 0, 80, and 120 mM NaCl salinity levels had higher TPC (10.09, 8.93, and 7.34%, respectively) than the control plants. Se treatment also increased the TPC of lemon verbena leaves by 7.79, 10.23, and 13.81% at salinity levels of 40, 80, and 120 mM NaCl compared to the control. Regarding flavonoids, both Se and N-Se treatments at all salinity levels, except for 160 mM NaCl, caused an increase in flavonoids (between 13 and 44%) compared to the control (Table 5).

**Figure 2:**
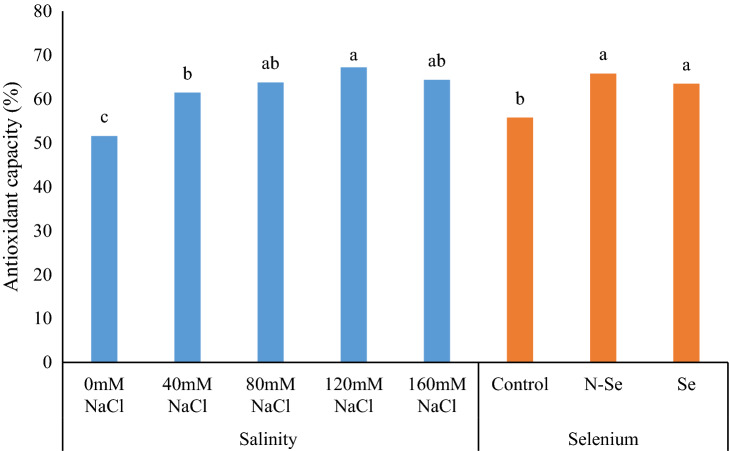
The influence of foliar sprays of selenium (Se) and nano-selenium (N-Se) on antioxidant capacity of lemon verbena (*Lippia citriodora*) plants under different salinity levels. Data represent means of three replication. Different letters in each column indicate significant differences at *p* < 0.05, according to Duncan’s multiple range test.

According to the ANOVA results, salinity and Se significantly affects total antioxidant capacity, while the interaction between them was insignificant (Table 1). In comparison to control plants, TAC was improved by 19.11, 23.55, 30.28, and 24.81% in plans subjected to 40, 80, 120 and 160 mM NaCl, respectively. Moreover, the application of N-Se and Se significantly improved the TAC by 17.99% and 13.84%, when compared with control plants (Fig. 2).

### Essential oil content

**Figure 3:**
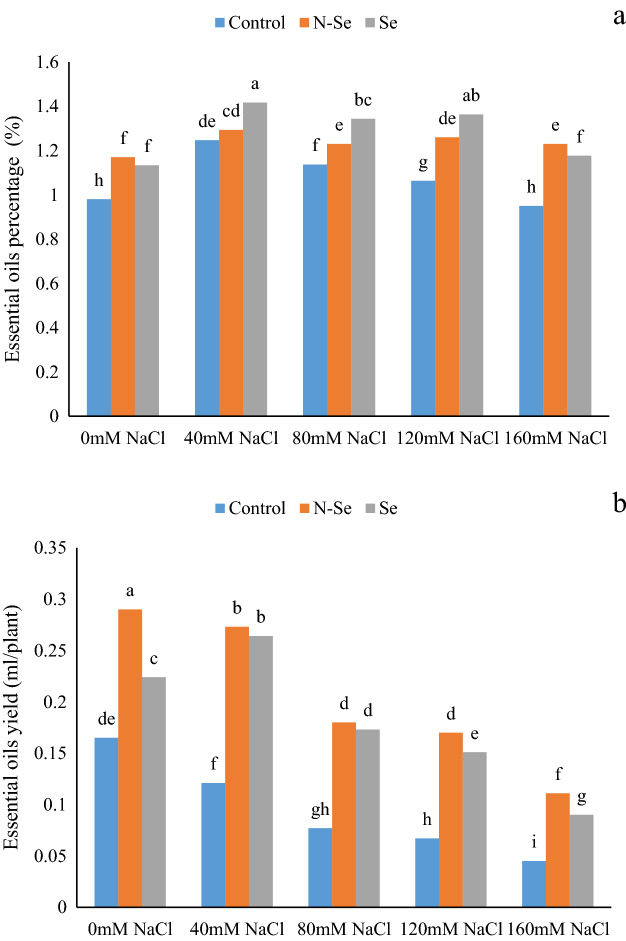
The influence of foliar sprays of selenium (Se) and nano-selenium (N-Se) on essential oils percentage (**a**) and essential oil yield (**b**) of lemon verbena (*Lippia citriodora*) plants under different salinity levels. Data represent means of three replication. Different letters in each column indicate significant differences at *P* < 0.05, according to Duncan’s multiple range test.

According to the ANOVA results, the percentage and the yield of the EOs were significantly affected by salinity, Se, and their interactions (Table 1). In the absence of Se application, salinity levels of 40, 80, and 120 mM NaCl caused an increase of 26.53, 15.30, and 8.16% of EOs content in lemon verbena, compared to the control, respectively (Fig. 3a). In contrast to these results, in control plants, the application of salinity levels caused a decrease of 26.66, 53.33, 59.39, and 72.72% of the EO yield compared to the non-stress plants (Fig. 3b). At the salinity levels of 0, 40, 80, 120, and 160 mM NaCl, N-Se treatment increased 19.38, 4.03 ns, 8.84, 18.86, and 29.47% of lemon verbena EO compared to the control, and Se treatment increased the content of lemon verbena essential oil by 15.30, 13.71, 18.58, 28.30 and 23.15% compared to the control, respectively (Fig. 3a). In addition, the application of 10 µM N-Se increased 75.75, 125.61, 133.76, 153.73, and 146.66%, respectively, of lemon verbena EOs yield at different salinity levels compared to the control. Moreover, applying 10 µM Se increased the EOs yield of lemon verbena by 35.75, 118.18, 134.67, 125.37, and 100.00% at different salinity levels compared to the control (Fig. 3b).

### Mineral contents

**Figure 4:**
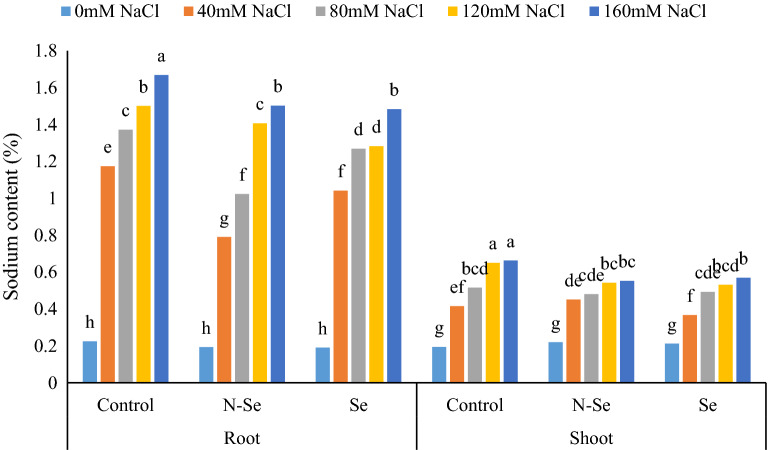
The influence of foliar sprays of selenium (Se) and nano-selenium (N-Se) on sodium content in shoots and roots of lemon verbena (*Lippia citriodora*) plants under different salinity levels. Data represent means of three replication. Different letters in each column indicate significant differences at *p* < 0.05, according to Duncan’s multiple range test.

The results showed that the interaction effect of salinity × Se on the Na^+^ content of roots and shoots was significant (Table 1). In control plants, salinity stress at the levels of 40, 80, 120, and 160 mM NaCl increased the Na^+^ content of root by 421.77, 509.60, 566.66, and 641.33%, and shoot Na^+^ content by 113.91, 165.46, 235.05 and 241.75% compared with the non-stressed plants. In non-stressed control plans, the Na^+^ content was not affected by N-Se and Se treatments. However, N-Se treatment at salinity levels of 40, 80, 120, and 160 mM NaCl caused a significant decrease (32.70, 25.38, 6.26, and 9.63%, respectively) of root Na^+^ compared to the control. Interestingly, Se treatment also reduced the Na^+^ content of roots by 11.41, 8.02, 14.66, and 10.84%, respectively compared to the control. Regarding the Na^+^ content of the shoots, N-Se and Se treatments only at 120 and 160 mM salinity levels caused a decrease in the Na^+^ content of the shoots compared to the control (Fig. 4).

**Figure 5:**
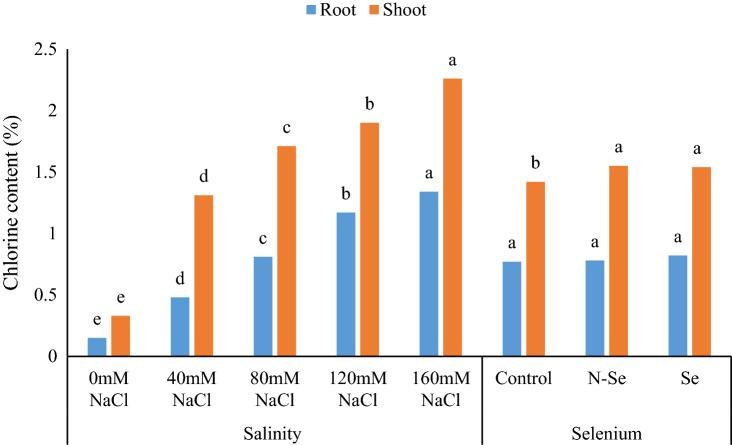
The influence of simple effects of salinity and selenium treatments on chlorine content in shoots and roots of lemon verbena (*Lippia citriodora*) plants. Data represent means of three replication. Different letters in each column indicate significant differences at *p* < 0.05, according to Duncan’s multiple range test.

The results showed that the effect of salinity on root Cl^−^ content and the effect of salinity and Se on shoot Cl^−^ content were significant. With increasing salinity levels, root and shoot Cl^−^ content increased and reached its highest level at 160 mM salinity. N-Se and Se treatments had no significant effect on root Cl^−^ content compared to the control. Nevertheless, both N-Se and Se treatments significantly (9.15% and 8.45%, respectively) increased the Cl^−^ content of shoots compared to the control (Fig. 5).

## Discussion

Salinity is one of the most serious environmental factors that can reduce the growth and performance of plants because most crops are sensitive to high salinity^34,56^. Lemon verbena, a medicinal plant, is also considered one of the plants sensitive to high concentration of NaCl, because salinity stress has deleterious effects on the biomass production of this plant^52^. In the present study, salinity stress caused a decrease in the growth parameters of lemon verbena plants. With the increase of the salinity level, the growth parameters of the plant decreased more strongly, so that the lowest plant height and SFW, SDW, RFW and RDW were observed at the most severe stress level (160 mM NaCl). In this regard, it has been reported that salinity stress negatively affects the growth and performance of medicinal plant species^29,36,52^. In the conditions of salinity stress, the normal metabolism of plant cells is disturbed, and as a result of excessive production of ROS, oxidative stress occurs in plants^25^. In these conditions, ROS damage chlorophyll, proteins, and cell membranes, and plants raise their antioxidant levels to deal with oxidative stress^19^. In the current research, some negative effects of salinity stress caused by NaCl were observed on the physiological processes of the lemon verbena plant. Increasing the amount of NaCl in the environment of lemon plant roots decreased chlorophyll and RWC and increased the content of proline, soluble sugar, MDA, LEL, H_2_O_2_, and antioxidant enzymes.

The results showed that foliar spraying of lemon verbena with 10 µM N-Se and Se increased the plant's growth parameters and RWC at different levels of salinity stress. Moreover, The LEL and accumulation of MDA and H_2_O_2_ in lemon verbena treated with N-Se and Se was lower than control plants, which indicates the reduction of oxidative damage caused by salinity in plants treated with Se. It has been reported that the accumulation of MDA and H_2_O_2_ under stress conditions has been related to oxidative damage^23^. Antioxidant defense system including enzymatic and Non-enzymatic components scavenge the ROS to provide plant tolerance against abiotic stress^19^. On the other hand, the results showed that the exogenous application of Se and N-Se increased the proline, soluble sugars, and total protein of lemon verbena plants under salinity stress conditions. The accumulation of proline and other osmolytes in plants under stress with the exogenous application of Se has been previously reported^27,43^. In the conditions of osmotic stress, proline has a special role in osmotic regulation, removal of free radicals, conservation of energy, protection of macromolecules, and in this way, it helps plants tolerate stress conditions^35^. In this study, accumulation of osmolytes in order to reduce oxidative damage, and improvement of plant growth in lemon verbena under salinity stress indicate the activation of defense mechanisms induced by the exogenous application of Se. In this regard, it has been reported that Se treatment by changing the physiological processes of the plant, such as the accumulation of protein and osmolytes, the improvement of the antioxidant capacity, the ion homeostastis, as well as improvement of the photosynthetic capacity has reduced the effects of stress in the plant and improves the plant growth under stress conditions^18,20,25,28^. Proietti et al.^40^ reported that the application of Se improves the tolerance of olive plants to drought stress by protecting cells from oxidative damage and regulating cell water status. In addition, it has been reported that the application of Se can increase the concentration of chlorophyll in plants by protecting chloroplast enzymes and increasing the biosynthesis of photosynthetic pigments^11,38^. In the current study, the use of Se treatment caused a significant increase in the antioxidant enzymes CAT and SOD and reduced the oxidative damage caused by salinity in the lemon verbena plant.

The production and biosynthesis of secondary metabolites in medicinal plants are strongly influenced by abiotic stresses and plant nutrition^1,53^. Salinity stress in the root zone reduces water absorption and causes nutrient imbalance in the plant, affecting the growth and development^24,45,54^. However, it may increase the biosynthesis of secondary metabolites in medicinal plants^53^. Thus, more studies are needed for depicting the role of salinity on production of secondary metabolites in aromatic plants. In the present research, applying salinity stress (in the absence of Se) at 40, 80, and 120 mM NaCl levels in lemon verbena medicinal plants increased the EOs percentage. There is much evidence of the positive effect of salinity stress on improving the biosynthesis of the essential oil of medicinal plants^5^. Nevertheless, some research also shows that this effect is not stable, and in some cases, secondary metabolites have been reduced under salt stress conditions^36^. In the present research, salinity stress (in the absence of Se) at all NaCl levels caused a significant decrease in the yield of the EOs in the lemon verbena medicinal plant. Similarly, a reduction of EOs yield in medicinal plants has been reported for lemon verbena^21^ and *Mentha canadensis* L^54^. under osmotic stress condition, which is consistent with the present research results. Since the yield of EOs is calculated as relation between extracted EO and the plant dry weight, therefore, a sharp decrease in dry matter due to salt stress led to a decrease in the yield of the EOs in salt stress treatments compared to the control.

Our results showed that the treatment of lemon verbena plants under salinity stress with 10 µM N-Se and Se increased the percentage and yield of EOs compared to the control plants. Similarly, to these results, Lee et al.^32^ reported that Se treatment increased the EOs content of the basil and balm plants by 2–3 times, compared to control. In another study, the application of 2 µM in nutrient solution and 5 µM Se foliar application significantly increased the production of the essential oil, hydroxycinnamic acids, phenolic compounds, and antioxidant activity of basil leaves^49^. Moreover, it is reported that Se treatments stimulated the biosynthesis of secondary metabolites in some species under environmental stress conditions^17,18^. Although the mechanism of selenium's action on plants' secondary metabolites is not well known, Se treatment may changes the expression of some specific genes related to the biosynthesis of secondary metabolites in plants^46^.

One of the most significant negative effects of salinity stress in plants is the increased absorption and accumulation of Na^+^ and Cl^−^ ions in plant tissues and, as a result, the ion toxicity of plant cells^24,50^. Ionic toxicity leads to some physiological and biochemical abnormalities, the production of free radicals, and nutritional imbalance in plant tissues^25,47,54^. The results of present research showed that the ionic balance in the roots and shoots of the lemon verbena plant was disrupted by salinity stress, so that salt stress led to a high accumulation of Na^+^ and Cl^ˉ^ in the plant tissues compared to the control. It is well known that salt stress tolerance in plants depends on their ability to limit the absorption and transfer of toxic ions from roots to shoots^45^. In the current research, under salt stress conditions, the application of 10 µM N-Se and Se caused a significant decrease in Na^+^ concentration in the roots and leaves of the lemon verbena plant. Results are in agreement with Jiang et al.^25^ documented that application of 1 and 5 µM Se decreased Na^+^ in the roots of corn plants under salinity stress. In mung bean plants under 100 mM salinity, applying 1 ppm of Se decreased the Na^+^ content of shoots and anthers by 35 and 10% compared to the control, but it had no significant effect on Cl^−^ content^28^. These results show that Se may interfere with the absorption and transport of Na^+^ in plants under salinity stress^28^,the mechanism of action of this process needs further investigation.

We also investigated the effect of salinity and Se treatments on some biomedical properties of lemon verbena. The biological properties of lemon verbena are related to the presence of some biological compounds with different chemical structures in the plant leaves^8^. It has been reported that lemon leaves are rich in phenolic compounds that play a significant role in the antiinflammatory and antioxidant properties of the plant^42^. The results showed that salinity stress significantly increased the phenolic and flavonoid compounds of lemon leaves compared to the control. Similar findings were reported in purple coneflower and grapevine plants^27,29^. The increased phenolic compounds in plants under stress protect the plant leaves against oxidative stress caused by salinity^29^. The results showed that the application of Se and N-Se improved lemon verbena leaf phenolic and flavonoid compounds under salinity stress conditions and thus increased its antioxidant capacity. The use of Se treatments in *Ocimum basilicum* L.^49^, *Artemisia annua*^17^ and *Brassica Juncea* (Handa et a, 2019) plants has increased the phenolic compounds, flavonoids, and anthocyanins, which is consistent with the results of the present research. These results may be related to the phenylalanine ammonia-lyase (PAL) activity, a key enzyme in the biosynthesis of phenolic compounds in plants^18^. These results show that Se treatments improve the antioxidant potential of the plant by increasing some secondary metabolites, and in this way, not only maintains the medicinal value, but also reduces the effects of salinity stress on lemon verbena plants.

**Figure 6:**
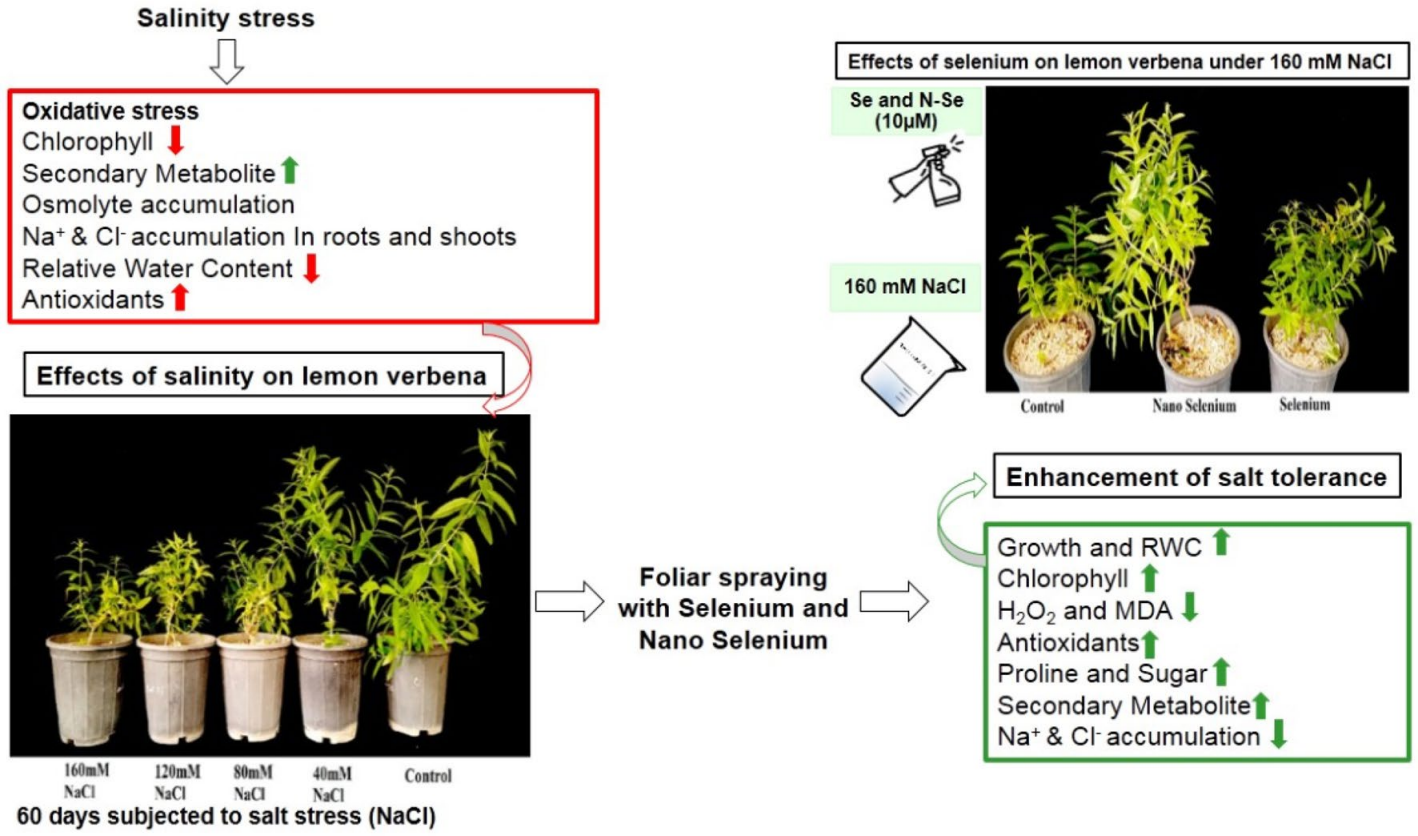
Schematic diagram for the effects of salinity stress created with NaCl and foliar application of selenium (Se) and nano-selenium (N-Se) on some physiological and biochemical aspects of lemon verbena medicinal plant. Salinity stress in lemon verbena plants led to oxidative stress and with the reduction of chlorophyll and relative water content, the accumulation of osmolytes and toxic ions led to the reduction of plant growth and productivity. The foliar application of N-Se and Se reduced the oxidative stress caused by salinity on the lemon verbena plant by improving plant water status and photosynthetic pigments, accumulation of soluble compounds such as proline and soluble sugar, improvement antioxidants and secondary metabolites and reduction of sodium accumulation in root and shoots.

In conclusion, the results revealed that foliar spraying of selenium at low concentration could diminish the harmful effect of salinity on lemon verbena, and this may be attributed to selenium effects on increasing pigments and RWC, accumulating osmolytes, biosynthesizing secondary metabolites, enhancing antioxidant capacity, and decreasing uptake and transferring Na + to the plant body at salinity condition (Fig. 6). In this regard, although both selenium and Nano- Selenium significantly reduced salinity effects on lemon verbena, Nano-Selenium remarkably improved lemon verbena’s growth and its phytochemical compounds. Hence, foliar spraying of selenium and Nano-Selenium on plants may serve as an effective strategy for mitigating salinity’s dire consequences, enhancing plant growth, and crop production. Considering the beneficial effects of selenium on plants and human beings, further investigations are required to recognize the potential of selenium in the plants production.

## Data Availability

The data sets analyzed during the current study are available from the corresponding author on reasonable request.
